# Monitoring Strategies of Earth Dams by Ground-Based Radar Interferometry: How to Extract Useful Information for Seismic Risk Assessment

**DOI:** 10.3390/s18010244

**Published:** 2018-01-16

**Authors:** Andrea Di Pasquale, Giovanni Nico, Alfredo Pitullo, Giuseppina Prezioso

**Affiliations:** 1 DIAN srl, 75100 Matera, Italy; info@dianalysis.eu; 2 Consiglio Nazionale delle Ricerche, Istituto per le Applicazioni del Calcolo, 70126 Bari, Italy; 3 Consorzio di Bonifica di Capitanata, 71121 Foggia, Italy; alfredo.pitullo@bonificacapitanata.it; 4 Dipartimento di Science e Tecnologie, Università degli Studi di Napoli “Parthenope”, 80133 Naples, Italy; pina.prezioso@uniparthenope.it

**Keywords:** ground-based radar, Synthetic Aperture Radar (SAR), Real Aperture Radar (RAR), SAR interferometry, earth dam

## Abstract

The aim of this paper is to describe how ground-based radar interferometry can provide displacement measurements of earth dam surfaces and of vibration frequencies of its main concrete infrastructures. In many cases, dams were built many decades ago and, at that time, were not equipped with in situ sensors embedded in the structure when they were built. Earth dams have scattering properties similar to landslides for which the Ground-Based Synthetic Aperture Radar (GBSAR) technique has been so far extensively applied to study ground displacements. In this work, SAR and Real Aperture Radar (RAR) configurations are used for the measurement of earth dam surface displacements and vibration frequencies of concrete structures, respectively. A methodology for the acquisition of SAR data and the rendering of results is described. The geometrical correction factor, needed to transform the Line-of-Sight (LoS) displacement measurements of GBSAR into an estimate of the horizontal displacement vector of the dam surface, is derived. Furthermore, a methodology for the acquisition of RAR data and the representation of displacement temporal profiles and vibration frequency spectra of dam concrete structures is presented. For this study a Ku-band ground-based radar, equipped with horn antennas having different radiation patterns, has been used. Four case studies, using different radar acquisition strategies specifically developed for the monitoring of earth dams, are examined. The results of this work show the information that a Ku-band ground-based radar can provide to structural engineers for a non-destructive seismic assessment of earth dams.

## 1. Introduction

Synthetic Aperture Radar (SAR) interferometry is a mature technique for the measurement of displacements, using both spaceborne, airborne and ground-based SAR sensors (e.g., [1,2,3]. It has been applied to monitor geological phenomena such as landslides [4,5,6,7], subsidence [8,9], volcanic activity [10] and infrastructure [11,12]. In this work we focus on the monitoring of earth dams, an example of infrastructure of extreme importance for agricultural practices and water resource management. As far as microwave scattering properties are concerned, earth dams are more similar to landslides than to concrete dams, since they are characterized by terrain surfaces. In the open literature, there are few papers covering this specific application where spaceborne SAR data have been used to measure earth dam displacements [13,14,15,16]. In [13], the earth dam was still not in operation and the observation of the dam surface by satellite was facilitated by the empty reservoir. In [14], the use of CosmoSkyMed X-band SAR images allowed measurement of displacements in the Mosul dam. However, spaceborne SAR interferometry (InSAR) does not seem appropriate for the monitoring of earth dams, except for vertical displacements due to subsidence. In fact, dam displacements are mainly horizontal as they are due to the water level changes in time. As a consequence, the fraction of these displacements along the satellite Line-of-Sight (LoS) is almost negligible. For this reason, GBSAR interferometric sensors seem more appropriate as they offer more degrees of freedom in terms of observating geometry and temporal baselines. Therefore, in this paper we focus on the use of a ground-based radar to measure also the horizontal deformations of earth dam surfaces and the vibrations frequencies of concrete structures connected to dam infrastructures, which require a high-frequency sampling of displacements. This can open new perspectives for the implementation of a protocol for the monitoring of earth dams based on the ground-based radar technology. The review papers [3,12] provide the current off-the shelf solutions for the measurement of displacements and vibration frequencies by means of ground-based radar interferometers. In particular, [17,18] present the current approach to monitor dam displacements using a GBSAR sensor.

In this work, we present the results obtained within the AIM-DAMS project, funded by the Apulia region, for the monitoring of four earth dams located in the Apulia and Campania regions, in Southern Italy. The aim of the project has been to demonstrate the applicability of GBSAR interferometry for the monitoring of the whole dam infrastructures, consisting of earth dam surface, bridges, chalice-shaped spillway and other concrete structures, and to extract information on their dynamic response useful for the seismic assessment.

All these quantities can be provided by a ground-based radar, which is a useful tool to perform a complete dynamic analysis of the dam behavior under the action of the safety earthquake [19,20]. According to the International Commission On Large Dams (ICOLD) recommendations, a safety earthquake coincides with the maximum believable earthquake for the location of the dam, which would affect the normal operation or even require to empty the reservoir in order to carry out reparations [19]. Unfortunately, this radar technology is currently used only for research purposes, but it is not of common use in engineering practice. 

The novelty of this work consists in new methodologies that have been developed for the radar data acquisition and visualization of results. In particular, new visualization tools have been developed to facilitate an in-depth analysis of displacement and vibration frequency measurements and to identify specific targets on the dam. Radar data have been collected using both Synthetic Aperture Radar (SAR) and Real Aperture Radar (RAR) acquisition modes. As the goal of this paper is to demonstrate that a ground-based radar can provide useful information to the sophisticated analysis tools needed for earthquake analysis, different acquisition strategies have been adopted to measure the dam displacements with simultaneous downstream and upstream radar measurements and specific data acquisition schemes for estimating different components of the displacement vector. 

The structure of the paper is as follows. Section 2 introduces the basic of ground-based radar sensors and estimation of displacements and vibration frequencies based on the radar interferometry technique. Section 3 summarizes the topographic measurement currently used to characterize the dynamic behavior of an earth dam. The four dams studied in this work are described in Section 4. Results are reported in Section 5. A few conclusions are drawn in Section 6.

## 2. Ground-Based Radar Interferometry: Overview and Estimation of Displacements and Vibration Frequencies

The ground-based radar interferometry technique relies on a radar system that observes the same scene from the same location at different times. The basics concept is an RAR system, a stepped-frequency continuous-wave (SF-CW) radar, that emits a continuous wave with different progressive frequencies within a given frequency band. The corresponding echoes, backscattered by the scene give rise to the raw data. A SAR system is obtained if the radar antennas are moved along a rail of finite length, changing their positions of a constant step. At each position along the rail, the SF-CW radar, transmits frequency increasing within bandwidth, and the corresponding echoes are collected. In the case of a SAR system, the raw data structure consists of a matrix with the number of columns, given by the acquisition positions along the rail, and rows, given by the transmitted frequencies. The Frequency-Domain Back-Propagation (FDBA) algorithm provides the exact focusing method of GBSAR. It performs a coherent sum, *S*, of the different frequency contributions for each radar position, corrected for the phase delay [21]:

(1) $$S\left(\rho ,\psi \right)=\frac{1}{M_{R}N_{R}}\sum_{n=1}^{N_{R}}\sum_{m=1}^{M_{R}}d\left(x_{n},f_{m}\right)\cdot \exp\left\{i4\pi \;f_{m}\frac{R}{c}\right\}$$

where *d(x_n_,f_m_)* is the raw data matrix, *x_n_* and *f_m_* are the acquisition position and transmitted frequency, respectively, *R* is the distance of each point in the scene to the center of the synthetic aperture, *c* is the speed of light and *S*(ρ,*ψ*) is the focused image given in polar coordinates. Although the FDBA provides the exact focused GBSAR image, its implementation is very heavy. Its computational load is given by *O*(*M_R_·N_R_·M_F_·N_F_*) with (*M_R_*,*N_R_*) and (*M_F_*,*N_F_*) being the dimensions of the raw data matrix and focused image, respectively. Different approximated solutions have been proposed in literature ensuring computational efficiency (e.g., see [21] and references therein). The Real Aperture Radar (RAR) has a range resolution of ∆*R* = 0.75 m. If the *TX/RX* antennas are sled along rail, a SAR image can be created with an azimuth resolution ∆*x*:

(2) $$\Delta x=\frac{\lambda R}{2L}$$

where *R* is the range distance and *L* is the rail length (in this case *L* = 2 m). In interferometric applications the radar system acquires a time series {*S_i_*, *i* = 1,…,*N*} of either coherent 1D radar profiles or 2D SAR images of the scene. Couples of interferometric radar data can be selected taking consecutive acquisitions or obtained at different times. In both cases, for each pair of radar data the interferometric phase is computed as follows:

(3) $$\Delta \phi_{1,2}=\mathrm{atan}\left\{S_{2}\cdot conj\left(S_{1}\right)\right\}$$

where *S*_1_ and *S*_2_ are the two coherent complex-values radar data acquired at times *t*_1_ and *t*_2_, respectively. The LoS displacement *D*_1,2_ of a point *P* in the scene, occurred in the time interval [*t*_1_, *t*_2_] is related to the interferometric phase ∆*ϕ*_1,2_ by the relationship:

(4) $$D_{1,2}=\frac{\lambda }{4\pi }\Delta \varphi_{1,2}$$

where *λ* is the radar wavelength (in this case *λ* = 18 mm). The precision of the displacement measurements depends on the accuracy of the phase measurements and can be a fraction of millimeter, if artifacts due to phase propagation in atmosphere are identified and corrected (e.g., see [22]. In the case of RAR interferometric applications, radar data acquired with the minimum sampling time, in the order of a fraction of second, are interferometrically processed to accurately define in time the deformation profile and to measure the vibration frequency spectrum and displacement rates, a useful information when this technology is applied to the study of dams. In contrast, in SAR interferometric applications the acquisition of SAR images requires a few minutes and the interferometric couples can be formed using a temporal baseline larger than the minimum time interval between the acquisition of two successive SAR images. The reason for this choice is related to the particular monitoring, continuous vs. repeated campaign, or to the need to optimize the interferometric processing (e.g., increase the interferometric coherence or reduce atmospheric phase delay effects).

The research described in this paper has been carried out using the IBIS-S/L sensor, a commercial radar with interferometric capability built by IDS. The system consists of a sensor module, a control PC, a power supply unit and data acquisition software. The IBIS sensor is a stepped frequency continuous wave radar that transmits an electromagnetic signal at a central frequency of 17.2 GHz (Ku band) with a maximum bandwidth of 300 MHz, corresponding to a range resolution of 0.75 m. 

## 3. Topographic Survey as a Support to the Advanced Processing of GB-SAR and RAR Data

This section is devoted to the advanced processing of radar data and the visualization of results. Two new methodologies are presented. The first one is to render GBSAR images, displacement and coherence maps over the dam surface. This is a key point when analyzing the radar measurements. The antenna pattern is used to identify the illuminated area on the dam surface and the concepts of spaceborne SAR data geolocation are used to map the dam geometry on the radar images [23]. The knowledge of the geolocated radar position allows estimation of the LoS geometric correction factor, which is a co-product of the proposed methodology. This gives a tool to merge two or more independent sets of displacement measurements collected at different radar installation sites. The second methodology provides an effective visualization of RAR data, with the displacement and vibration frequency measurements displayed in a way that makes easier the identification of targets with similar dynamic properties.

### 3.1. Rendering of GBSAR Displacement Maps

**Figure 1:**
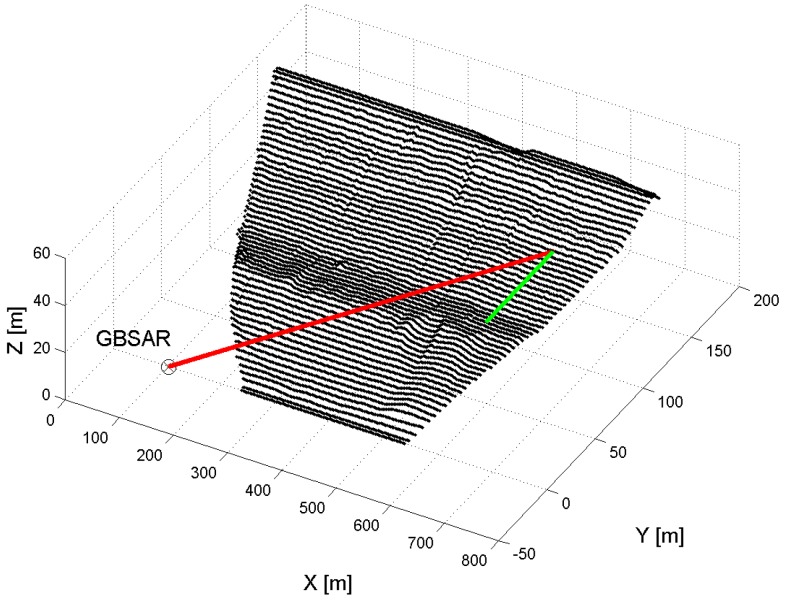
Geometry for the rendering of GBSAR displacement maps over a Digital Elevation Model (DEM). The range and displacement vectors are shown in red and green, respectively.

The rendering of GBSAR images, displacement and coherence maps requires a Digital Elevation Model (DEM) of the dam and the coordinates of the radar installation site, both given in the same reference system (e.g., see [24] and references therein). Let us denote with $r_{P}=\left(x_{P},y_{P},z_{P}\right)$ the Cartesian coordinates of a point of the DEM mesh, $r_{R}=\left(x_{R},y_{R},z_{R}\right)$ the coordinates of the center of rail and α the orientation angle of the rail measured with respect to the x-axis. Figure 1 shows an example of dam mesh, with the Cartesian reference system, the points $r_{P}$ on the mesh and $r_{R}$ at the center of the rail, and the orientation angle. Furthermore, the range vector $r_{P}-r_{R}$, as well as the displacement vector, d, supposed to be horizontal, are shown in red and green, respectively. Each pixel on the GBSAR images is defined in terms of the range distance R and the azimuth angle ψ, which is defined as the angle between the vertical plane perpendicular to the rail and containing its center, and the one containing both the center of the rail and a point on the dam mesh. To reduce the risk of ambiguities, i.e., of different points on the mesh having the pair of (*R*, *ψ*) coordinates, and so corresponding to the same pixel on the SAR image, a further angle is defined between two planes, both having the direction set by the rail as the rotating axis. The first plane has a tilt angle with respect to the horizontal plane given by the elevation angle *ϑ* of the radar; the latter is identified by the point. The angle between these two planes is defined as vertical angle *ξ*. Hence, for each point on the DEM mesh, the range distance *R*, the azimuth and vertical angles, *ψ* and *ξ*, are computed, respectively, as:

(5) $$R=\sqrt{{\left(x_{P}-x_{R}\right)}^{2}+{\left(y_{P}-y_{R}\right)}^{2}+{\left(z_{P}-z_{R}\right)}^{2}}$$

(6) $$\psi =\arcsin\left(\frac{x_{P}\cos\alpha -y_{P}\sin\alpha }{R\cdot \sqrt{\cos^{2}\alpha -\sin^{2}\alpha }}\right)$$

(7) $$\xi =\arctan\left(\frac{z_{P}-z_{R}}{R}\right)$$

**Figure 2:**
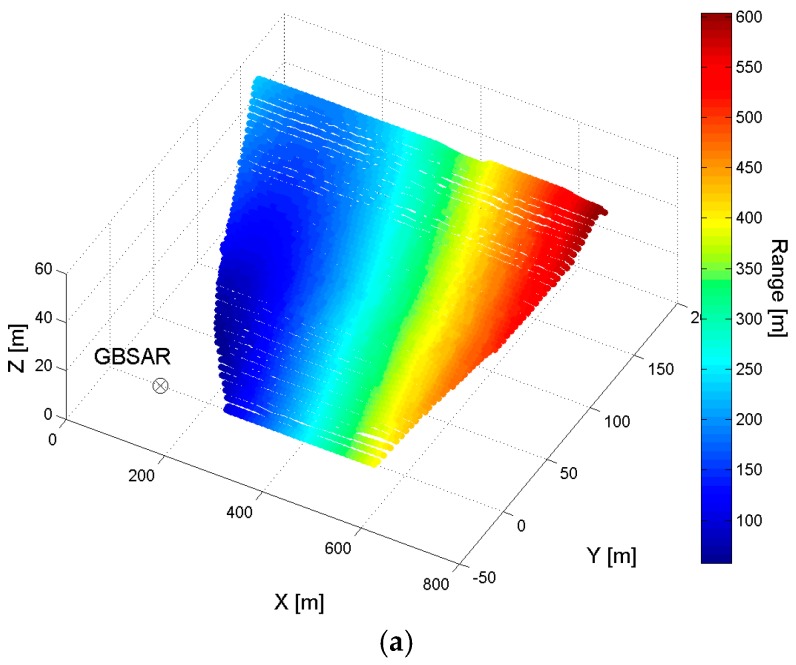

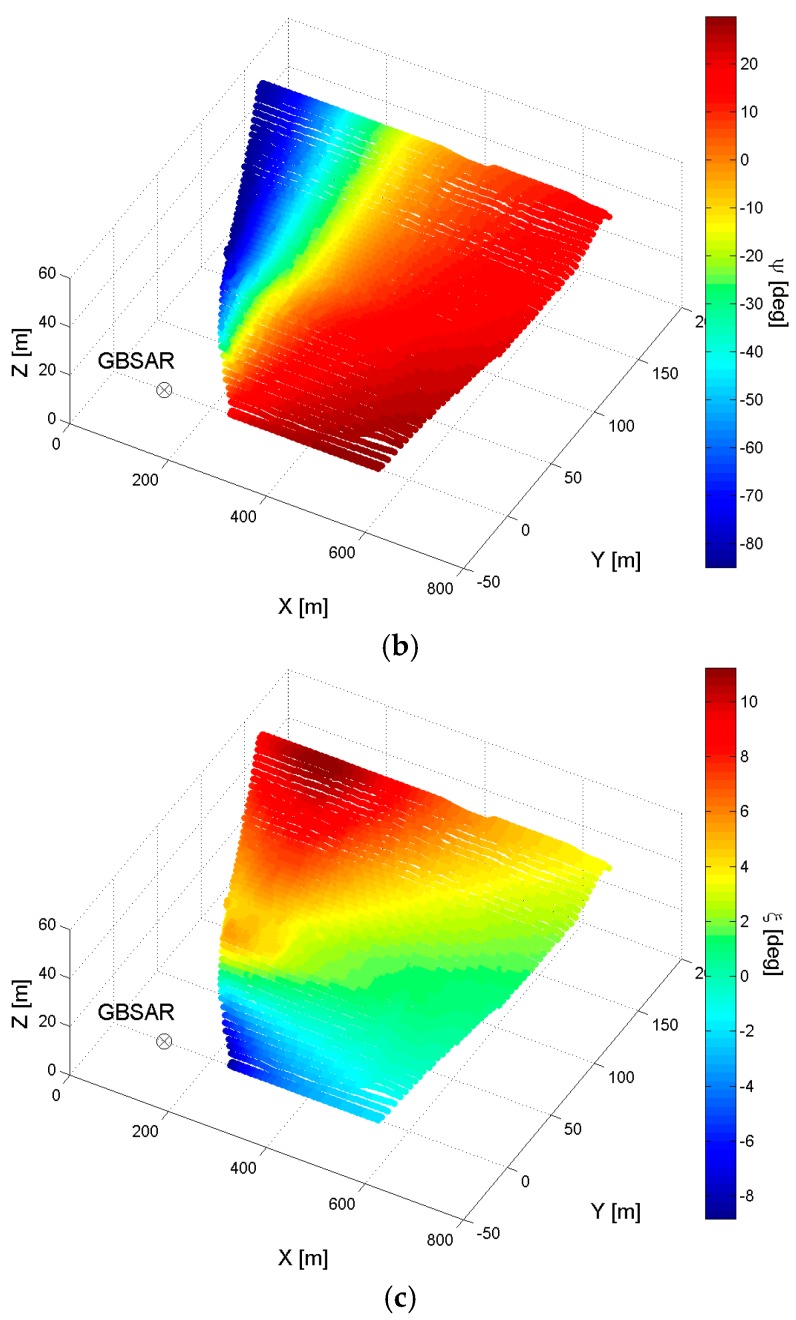
(**a**) Range distance *R*; (**b**) azimuth angle *ψ*; (**c**) vertical angle *ξ*.

Figure 2 displays the values of *R*, *ψ* and *ξ*, computed for each point of the dam mesh for a given position and GBSAR acquisition geometry (α,ϑ). The comparison of *ψ* and *ξ* angles with the horizontal and vertical angular widths of the antenna main lobe, respectively, allows identification of the area on the dam surface illuminated by the radar system and to which the measurements refers. This is a useful information when merging data acquired by different GBSAR systems, with slightly different installation sites with respect to the dam.

### 3.2. Estimation of the Line-of-Sight Geometric Factor 

The knowledge of the LoS geometric allows derivation of the displacement vector from measured LoS displacements. This requires the computation of the expected main direction of displacements, or at least to make a hypothesis on this direction. In the case of dams, surface displacements are mainly due to the water level changes in time and so we can assume that they are mainly horizontal displacements. Amplitude of displacements is unknown and can change over the dam surface. The knowledge of the radar location and acquisition geometry is also needed. The geometric factor *F*_LoS_ is computed as:

(8) $$F_{\mathrm{LOS}}=\frac{R}{\cos\eta \cdot \left(x_{P}-x_{R}\right)+\cos\zeta \cdot \left(y_{P}-y_{R}\right)}$$

where the unit vector of horizontal displacements is given in terms of direction cosines as $n_{disp}=i\cdot \cos\eta +j\cdot \cos\zeta$. 

**Figure 3:**
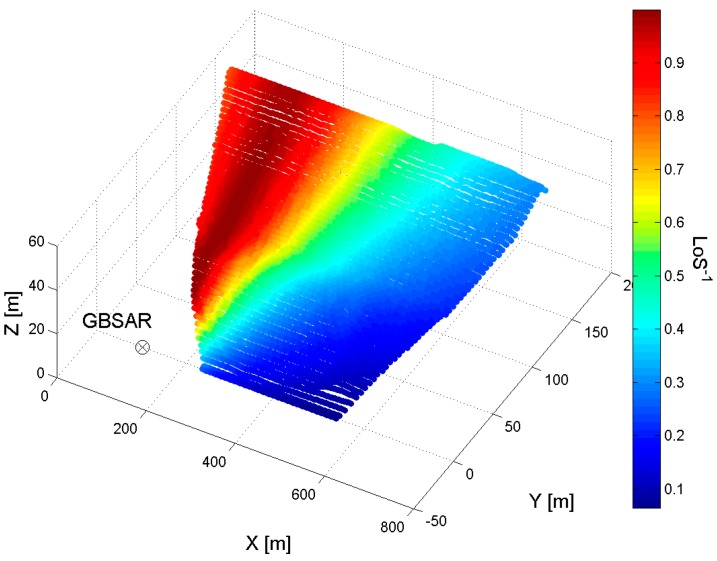
LoS factor spatial distribution over the dam mesh.

**Figure 4:**
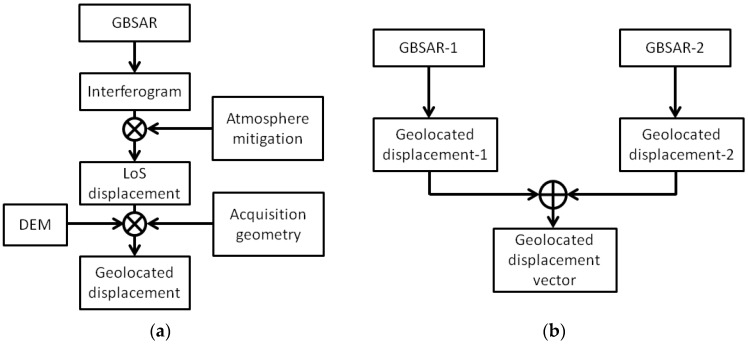
Sketch of the proposed methodology for the processing of ground-based SAR data: (**a**) geolocation, correction for the LoS factor and 3D rendering of displacement measurement; (**b**) merging of displacement measurement for the estimation of the geolocation displacement vectors.

Figure 3 shows an example of LoS factor rendered over the dam mesh, in the case of measurements acquired with antennas having a main lobe of 29 degrees in azimuth and 25 degrees in elevation. The correction of LoS displacement measurements with the LoS correction factor maps, provides the horizontal displacements of dam surface. Figure 4 summarizes the step of the methodology. 

**Figure 5:**
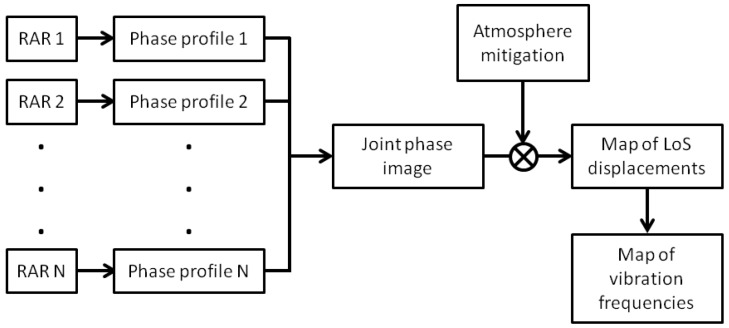
Sketch of the proposed methodology for the processing of ground-based RAR data.

Figure 5 summarizes the methodology used to process and visualize RAR measurements.

### 3.3. Visualization of RAR Displacement and Vibration Frequency Measurements 

The use of RAR data for the measurement of displacements and vibration frequencies of bridges and vertical structures is well detailed in the literature ([12] and references therein). Time series of displacements, and the corresponding frequency spectra are computed in a few key points, usually identified by corner reflectors. However, when using a ground-based RAR system to measure displacements and vibration frequencies of structural elements of an earth-filled dam, such as the chalice-shaped spillway or bridges and retaining walls, this approach cannot be easily applied since the access to these structures is quite difficult. Furthermore, a thorough analysis of data structural behavior of whole structure requires a visualization of displacements and vibration frequencies of all targets in the scene to compare their dynamical behavior. The visualization tools, used in this paper, show the displacement measurements as a 2D matrix where for range pixel, displacements are plotted vs. time. Analogously, the power spectrum is shown as a 2D matrix, where for each range pixel the amplitude spectrum is plotted vs. the vibration frequency. In this way, the coherent displacements of adjacent targets can be easily identified.

## 4. Dam Characteristics and Monitoring Methodology 

This work focuses on four earth-filled dams- located in the Apulia and Campania regions, southern Italy- and managed by the Consorzio di Bonifica di Capitanata. The following dams have been studied:

(1) Occhito, on the Fortore River. It was built between 1958 and 1966 and started to operate in 1972. It has one of the largest dam basins in Europe. The dam is located on the northern part of the Apulia region, in the districts of Carlantino and Celenza Valfortore, on the border between Apulia, Campania and Molise regions;
(2) Capaccio, on the Celone River. It was built between 1992 and 1997 and started to operate in 2000. It is located in the district of Lucera, on the northern part of the Apulia region;
(3) Marana Capacciotti, on the Ofanto River. It was built between 1969 and 1976, and started to operate in 1987. It is located 13.5 km south-west of the Cerignola city, on the northern part of the Apulia region, precisely;
(4) San Pietro, on the Osento River. It was built between 1958 and 1966, and started to operate in 1972. It is located in Aquilonia district, on the western part of the Campania region, on the border with the Basilicata region.

The RAR data have been used to measure the displacements and vibration frequencies of dam concrete structures. Instead, the SAR data have been acquired to measure the surface displacements of the earth-filled dam. In particular, three different acquisition methodologies have been used to collect the SAR data. The simplest acquisition methodology relies on only one GBSAR system and can provide the LoS component of the surface displacements. The second methodology uses two GBSAR systems, installed at two almost symmetrically downstream sites. It can be used to derive two displacement maps covering the whole dam surface. In the overlapping area of the two maps, the displacement vector of the surface can be measured. This methodology relies on the hypothesis that the displacement vector is almost horizontal as it should be in the case of dam deformation caused only by theater level temporal change. The third acquisition methodology uses two GBSAR systems located almost symmetrically on the upstream and downstream sides of the dam. It can be used to simultaneously measure LoS displacements of the upstream and downstream dam surfaces. 

**Figure 6:**
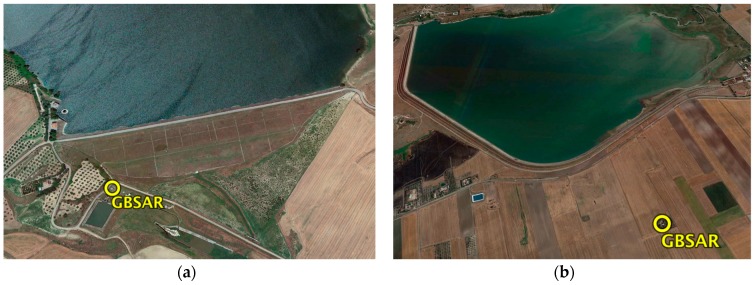

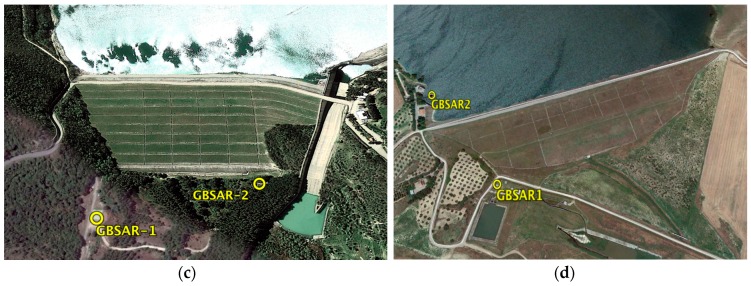
Google© snapshots of (**a**) Marana-Capacciotti dam on the Ofanto river, Cerignola; (**b**) Capaccio dam on the Celone stream, Lucera; (**c**) Occhito dam on the Fortore river, Carlantino and (**d**) San Pietro dam on the Osento stream, Aquilonia. The GBSAR installation sites are also shown.

Figure 6 displays the Google© snapshots of the four dams with the installation sites of SAR acquisitions. In particular, Figure 6a,b show, respectively, the Marana Capacciotti and Capaccio dams. The first acquisition methodology has been used to measure LoS displacements of the two dams using only one GBSAR. Figure 6c shows the Occhito dam where the second methodology has been used. The location of the two GBSAR systems is also reported. Finally, Figure 6d shows the San Pietro dam with the location of the two GBSAR system used to measure the LoS displacements using the third methodology.

## 5. Results

In this section, we summarize the results obtained within the AIM-DAMS project for the monitoring of earth-filled dams by using GBSAR and RAR interferometry. These results are organized in two different sub-sections, each one devoted to a different data acquisition setup and processing methodology, with the aim to provide quantitative information on the behavior of earth dams. Section 5.1 deals with the problem of estimating the displacement amplitude of dam surfaces. The methodology for computing the geometric factor, described in Section 3.2, is applied to correct the LoS displacement maps. The results presented in Section 5.1 provide a protocol to generate displacement maps by merging data acquired at different installation sites, and in repeated campaigns. These maps can be useful to assess the output of finite element models used to study the dam behavior under different external conditions and to provide to structural engineers information about the stiffness of the dam body. Finally, Section 5.2 focuses on the measurement of dynamical properties of dam concrete structures. The main results refer to the measurement of displacement and vibration frequencies of a pedestrian bridge, a chalice-shaped spillway and a pillar. 

### 5.1. Visualization of Dam Surface Displacements 

**Figure 7:**
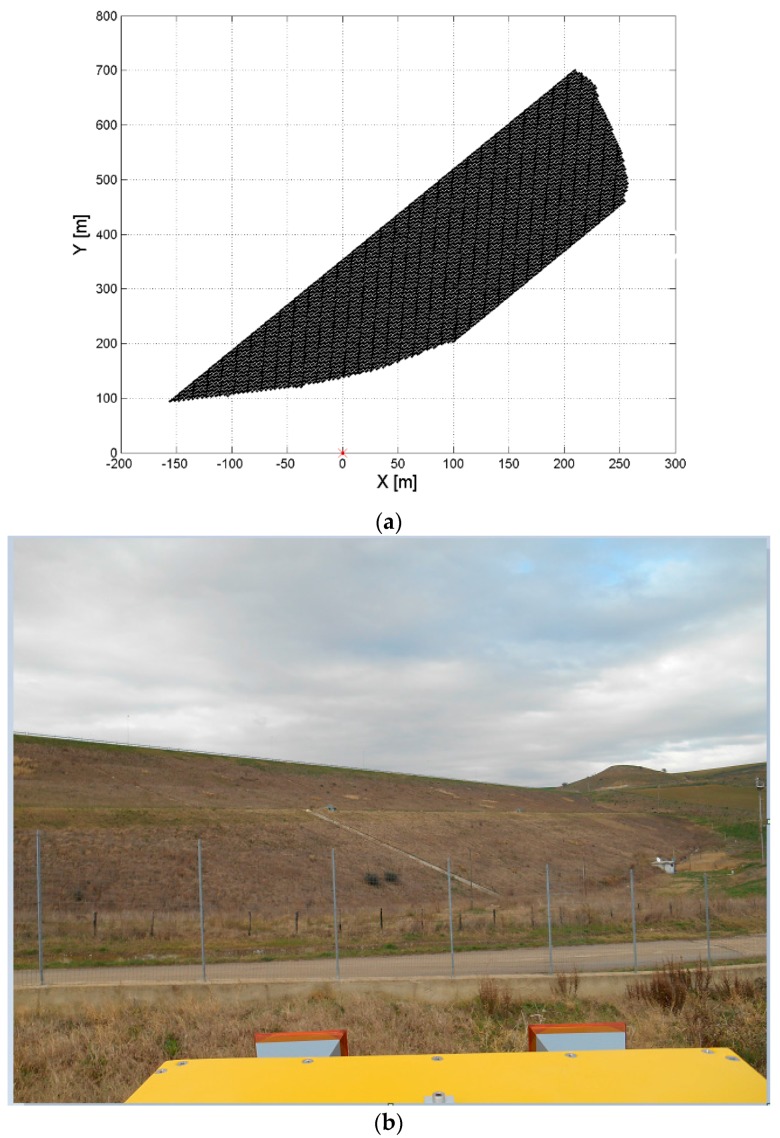
Acquisition geometry at the Marana-Capacciotti dam: (**a**) Map of the dam downstream surface re-projected in the Cartesian reference system centered at the GBSAR rail center and with the x-axis given by the rail direction. The GBSAR is located at the position (*x* = 0, *y* = 0); (**b**) photo of the dam surface taken from the GBSAR installation site.

**Figure 8:**
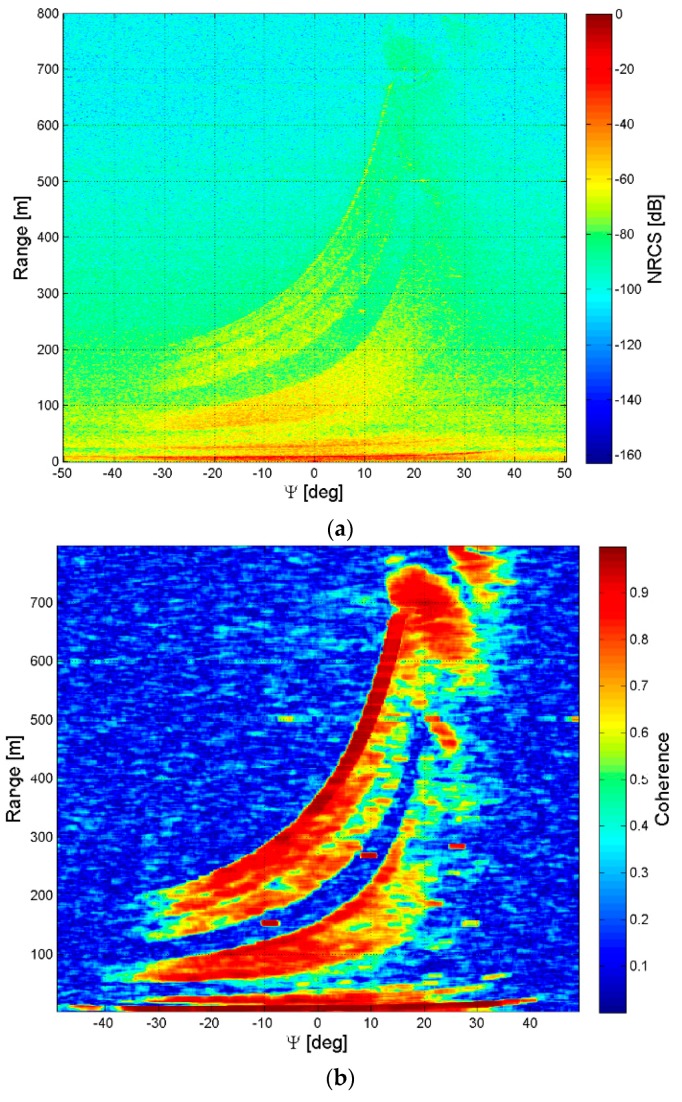

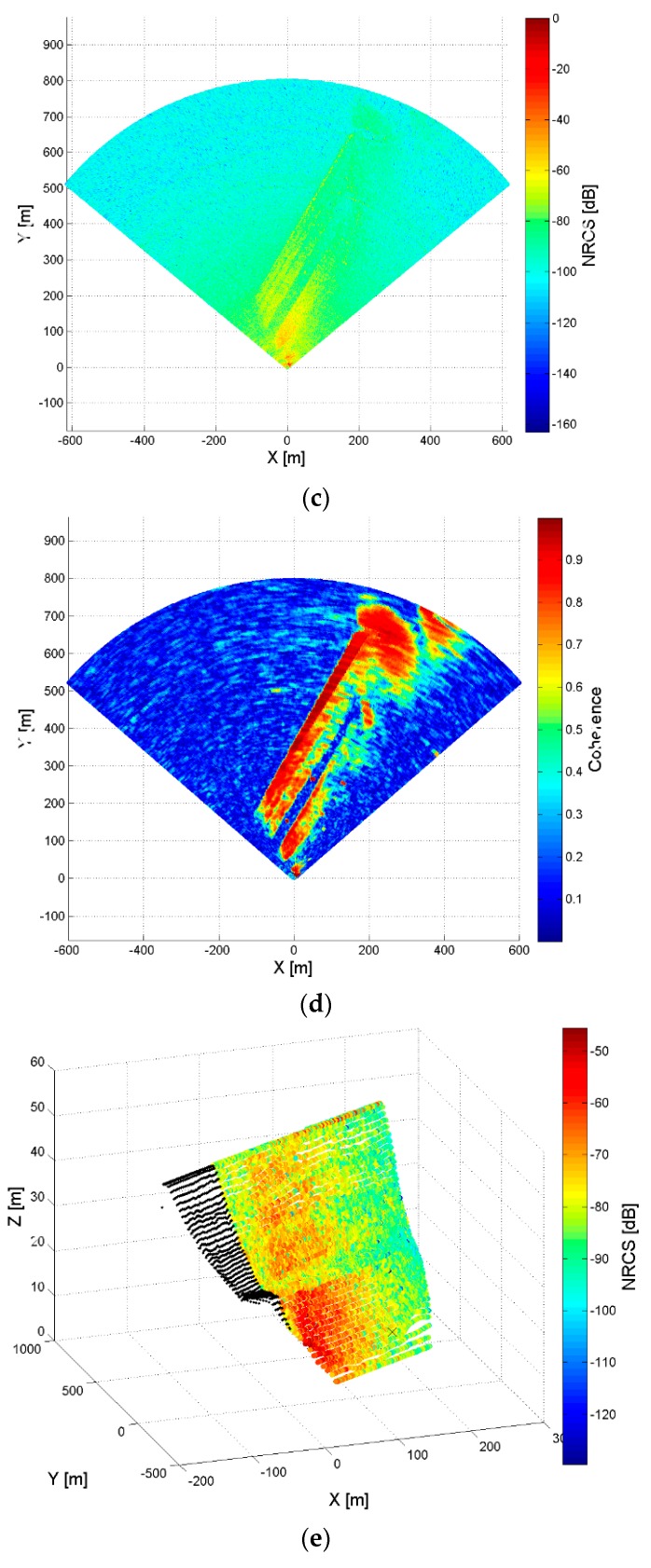

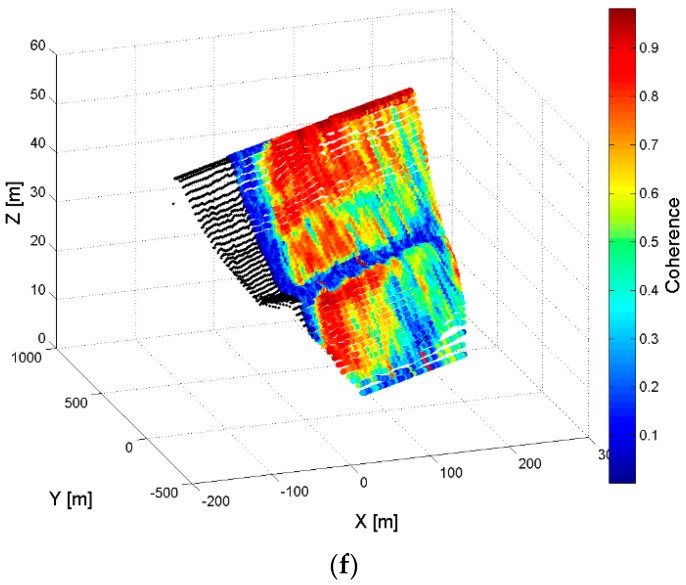
Marana-Capacciotti dam, Cerignola. NRCS and interferometric coherence displayed in radar coordinates (**a**,**b**), Cartesian coordinates (**c**,**d**) and rendered on a Digital Elevation Model (DEM) of the dam (**e**,**f**).

In this section, we describe different ways to visualize the GBSAR information on dams. The extension of the scene observed by the GBSAR is set by the maximum range distance and antenna beamwidth. Figure 7 shows the planimetric projection of the Digital Elevation Model (DEM) of the Marana-Capacciotti dam surface, and a photo of the dam taken from the GBSAR location site. The planimetric resolution of the DEM is 3 m. The DEM coordinates are given in the local Cartesian reference system with the x-axis oriented along the radar rail. The GBSAR is located at the origin of this reference system. Figure 8 shows the Normalized Radar Cross Section (NRCS) images and coherence maps, of the Marana-Capacciotti dam. The maximum range distance has been setup to *R* = 800 m with an antenna beamwidth *ψ* ∈ [−51, 51] degrees. Figure 8a,b display the GBSAR image and coherence map in radar coordinates (*R*, *ψ*). This representation is particularly useful during all the processing steps of radar data and the identification and mitigation of atmosphere phase delay effects. However, this kind of visualization can make difficult the analysis of results as the dam geometry is transformed to the radar coordinate system. 

**Figure 9:**
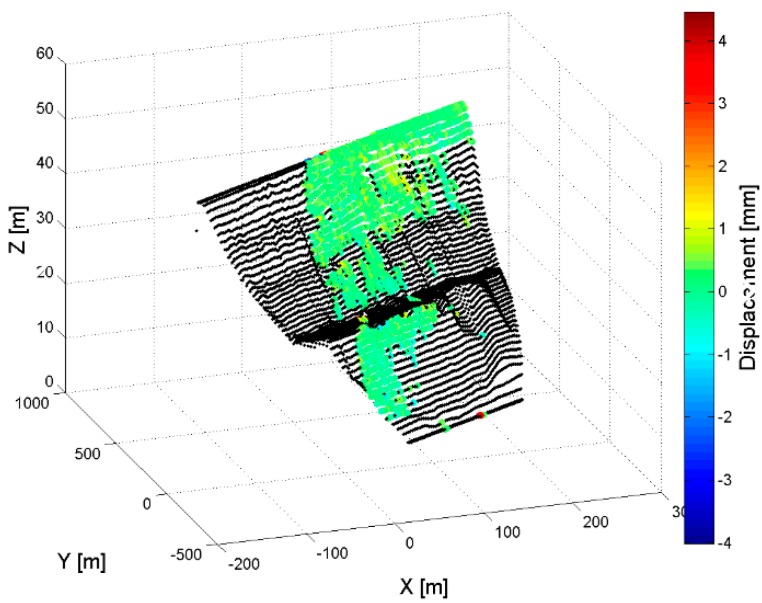
Marana-Capacciotti dam, Cerignola. Amplitude of the horizontal displacement map represented over the dam surface.

Figure 8c,d display the GBSAR information in the same Cartesian reference system used to display the dam geometry in Figure 7a. The comparison of Figure 7a and the NRCS image and coherence maps in Figure 8a,b help to easily recognize the dam body within the scene. In particular, the coherence map clearly shows the coherent radar signal scattered from the hill beyond the dam (see also the photo in Figure 7b). The rendering of the same information on a DEM of the dam surface is reported in Figure 8e,f. In particular, the coherence map in Figure 8f identifies the portion of the dam that appears coherent in the SAR images collected with that specific acquisition geometry. Accurate displacement measurements can be provided only in this coherent region, or in a smaller portion of it if SAR images acquired with a large temporal baseline are processed. Figure 9 displays an example of displacement map rendered on the dam surface and corrected for the LoS geometric factor. This kind of 3D visualization is particularly appropriate to extract information on dam structural properties. However, it requires a DEM of the dam structures, which could be unavailable at the moment of the radar acquisition. 

As a sub-optimal solution, dam structural properties can be derived using the visualization in Cartesian coordinates of Figure 8e,f. This visualization loses information on target heights as all targets are projected on the horizontal plane used to visualize GBSAR data. 

**Figure 10:**
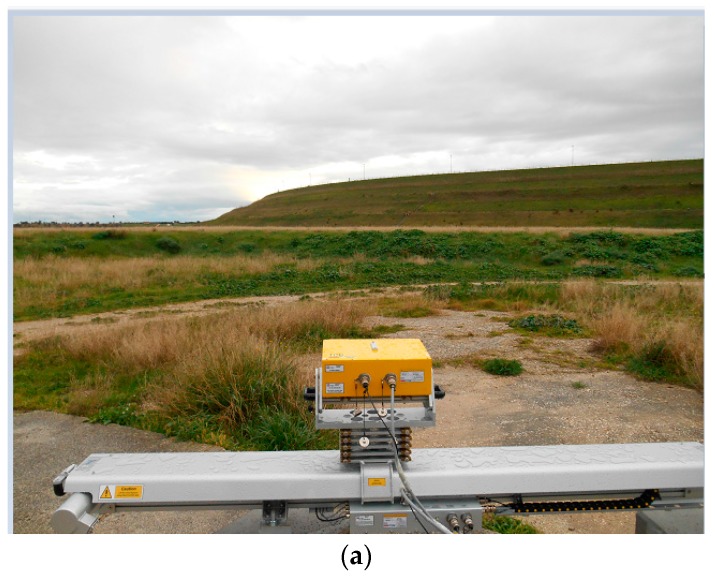

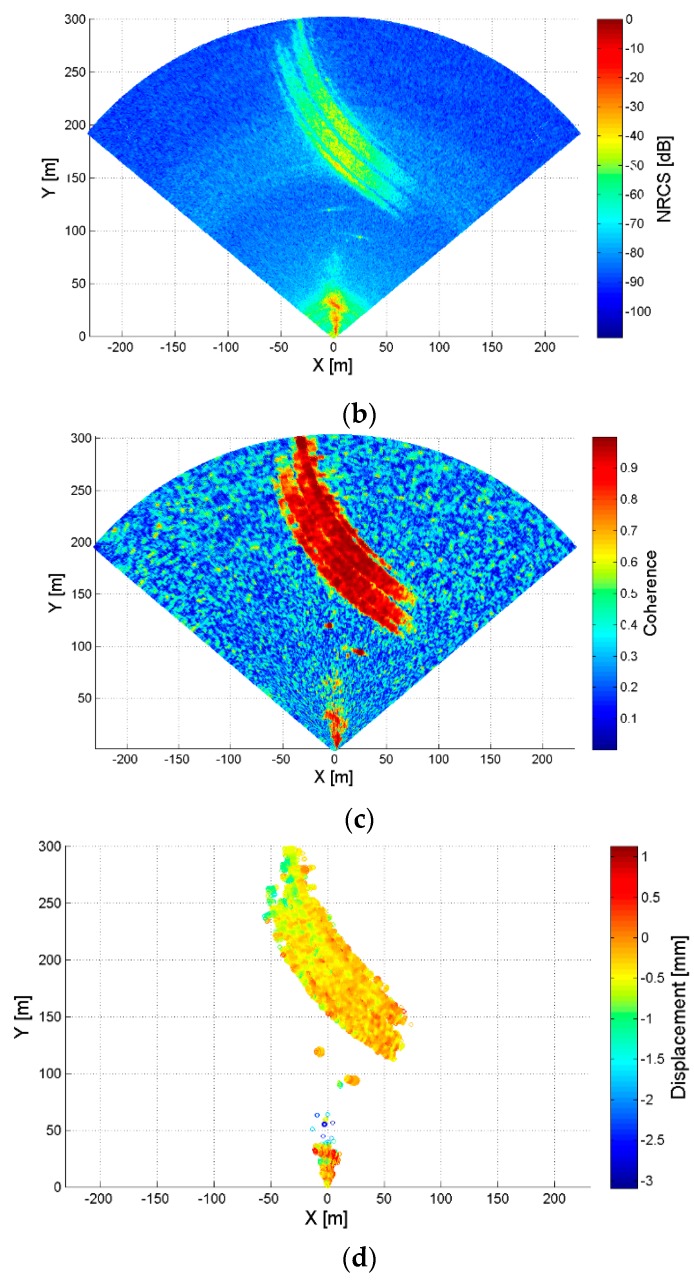
Capaccio dam, Lucera. (**a**) Photo of the dam as observed by the GBSAR position; (**b**) NRCS; (**c**) interferometric coherence and (**d**) displacement map in Cartesian coordinates.

In case of a simple acquisition geometry, where the GBSAR is installed on an almost perfect flat area, the visualization of GBSAR data in a Cartesian reference system can still be useful to distinguish targets in height. Figure 10 refers to the monitoring of the Capaccio dam. The downstream side of the dam is perfectly flat and the GBSAR has been installed in front of the dam, looking at the curved structure of the dam (see Figure 10a). The geometric structure of the dam is clearly identified in the NRCS images and coherence map, reported in Figure 10b,c. Due to the particular acquisition geometry, targets located at increasing distances, measured in the horizontal plane, are also located at increasing heights above the horizontal reference plane used to visualize the GBSAR data. As a consequence, an interpretation of GBSAR data in terms of the 3D structure of the dam can be done also without rendering radar data on the DEM of the dam surface.

**Figure 11:**
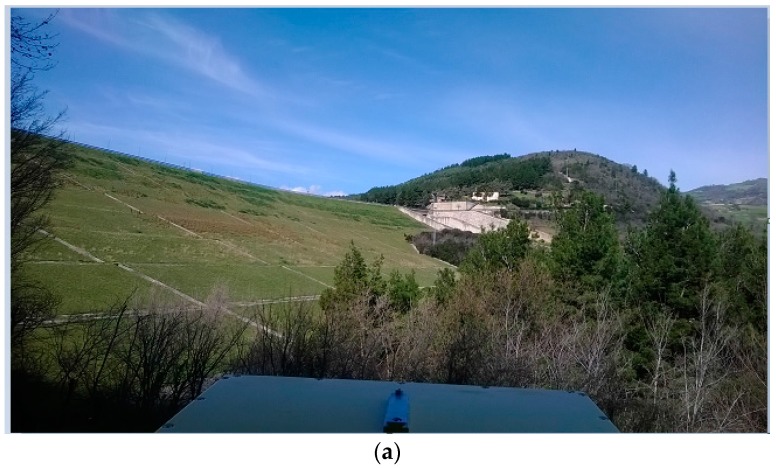

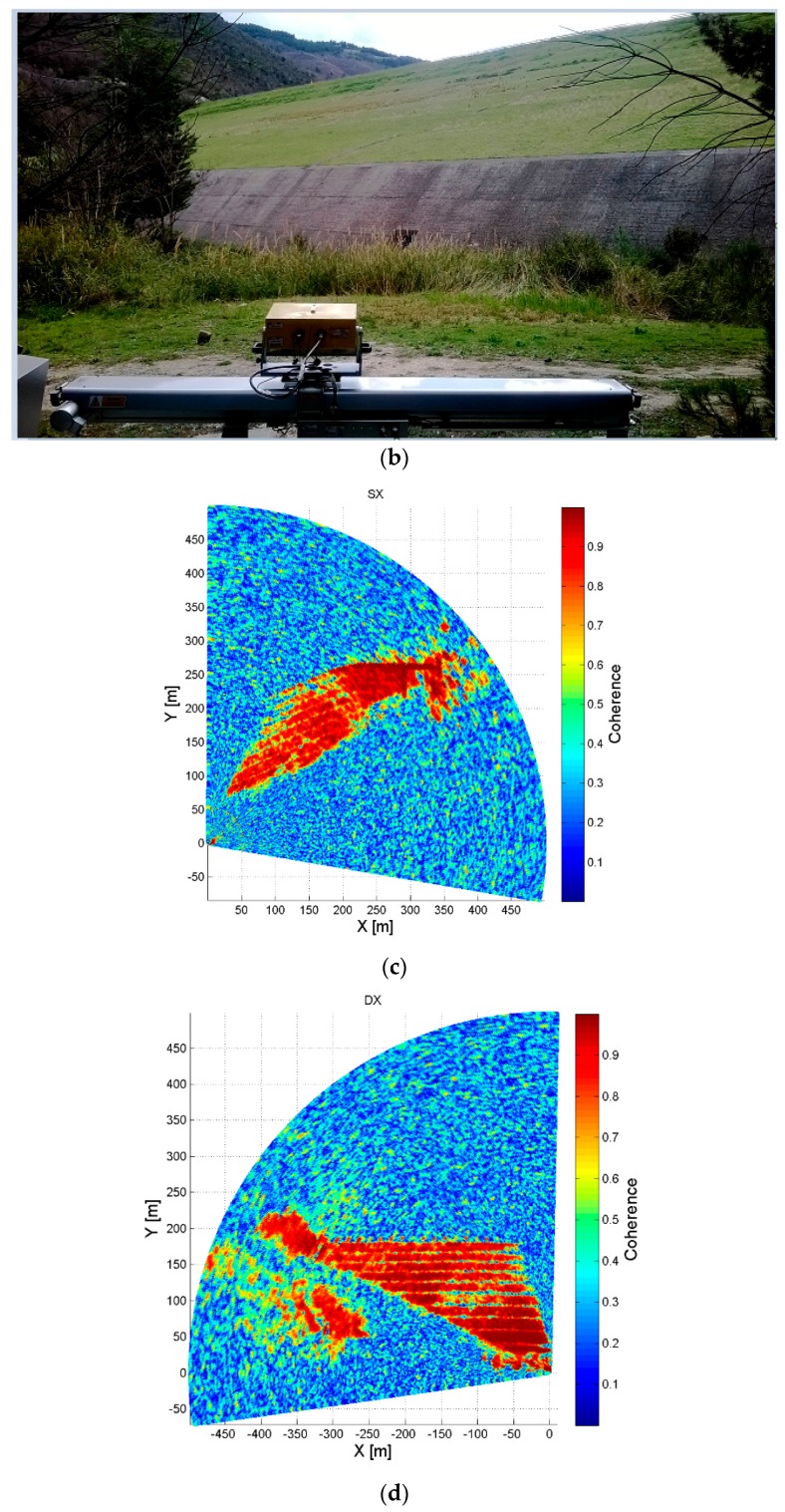

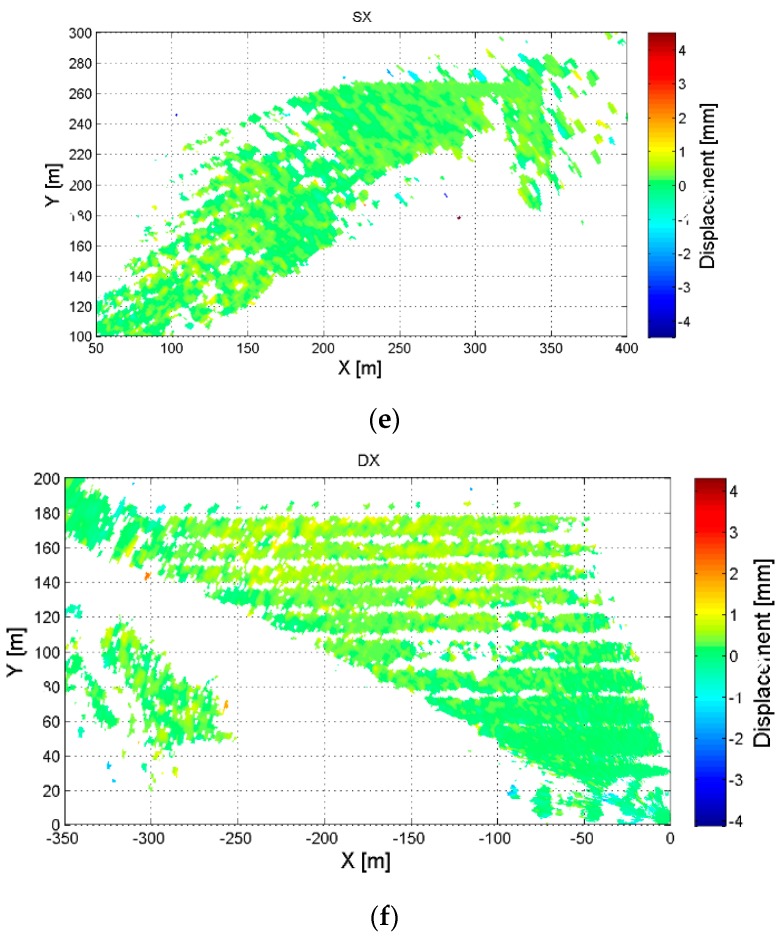
Occhito dam, Carlantino. (**a**,**b**) photo of the dam as observed from the GBSAR positions; (**c**,**d**) interferometric coherence (**c**,**d**); (**e**,**f**) displacement maps in Cartesian coordinates. Data have been collected by two GBSAR systems installed on the left (SX) and right (DX) downstream sides of the dam, respectively.

As a further example, Figure 11 reports the visualization of coherence and displacement maps of the Occhito dam. The simple acquisition geometry of the GBSAR system allows inference of the 3D structure of the dam from the visualization of GBSAR data in the local Cartesian reference system. In this case, GBSAR data have been acquired from two monitoring stations located on the left and right down-stream sides of the dam. This particular observation scheme can avoid the computation of the LoS correction factor as two independent measurements of the terrain displacements can be obtained for the portion of the dam surface covered by the overlap of the two antenna footprints. If the displacement vector of the dam surface can be considered horizontal, as in case of displacement due to the water level changes in time only, the two components of the displacement vector are estimated from the LoS displacements measured at the two radar positions.

**Figure 12:**
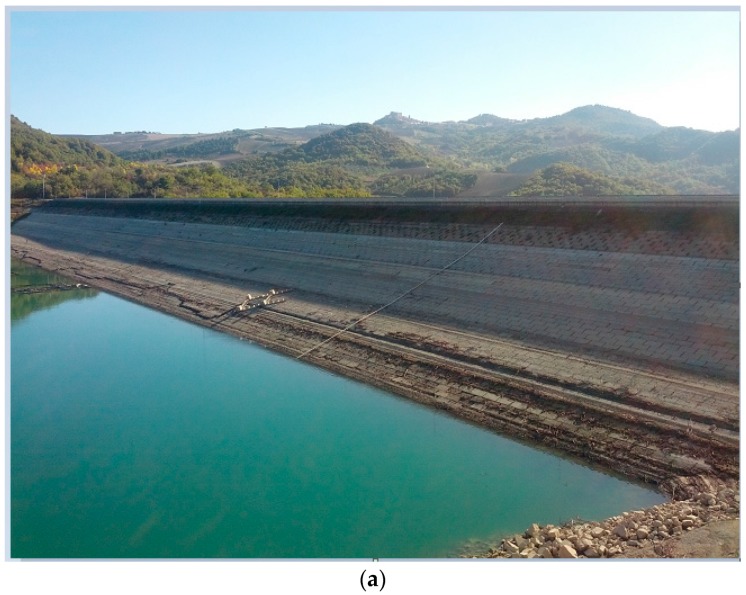

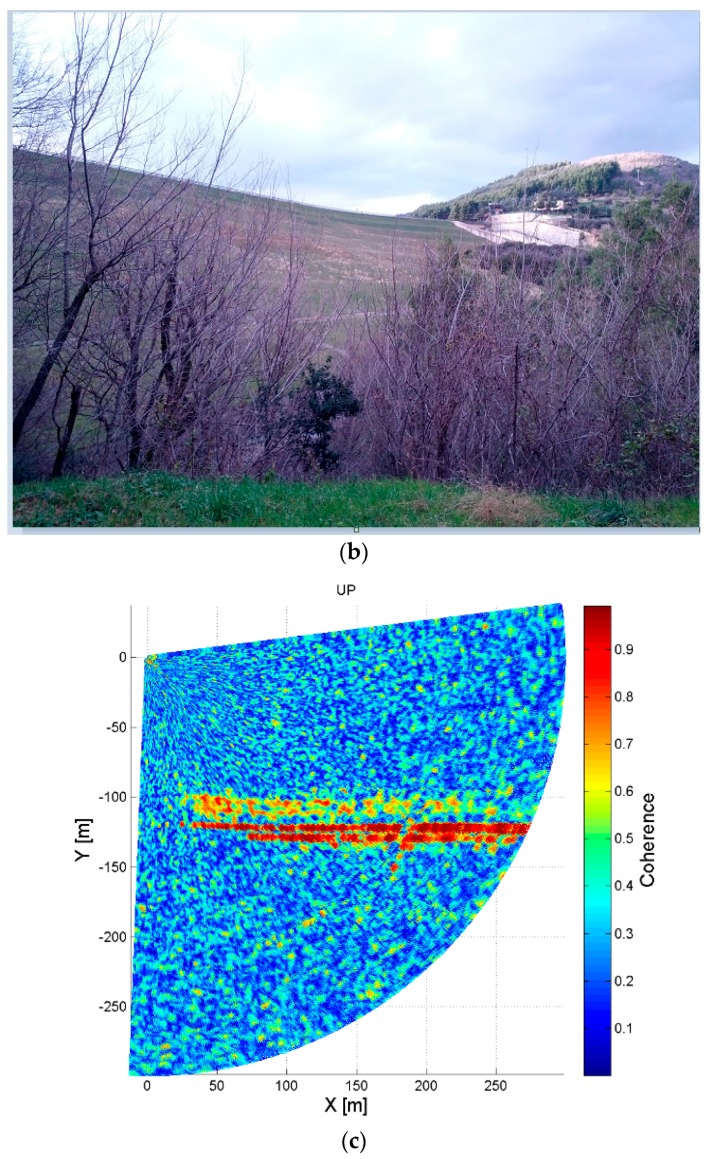

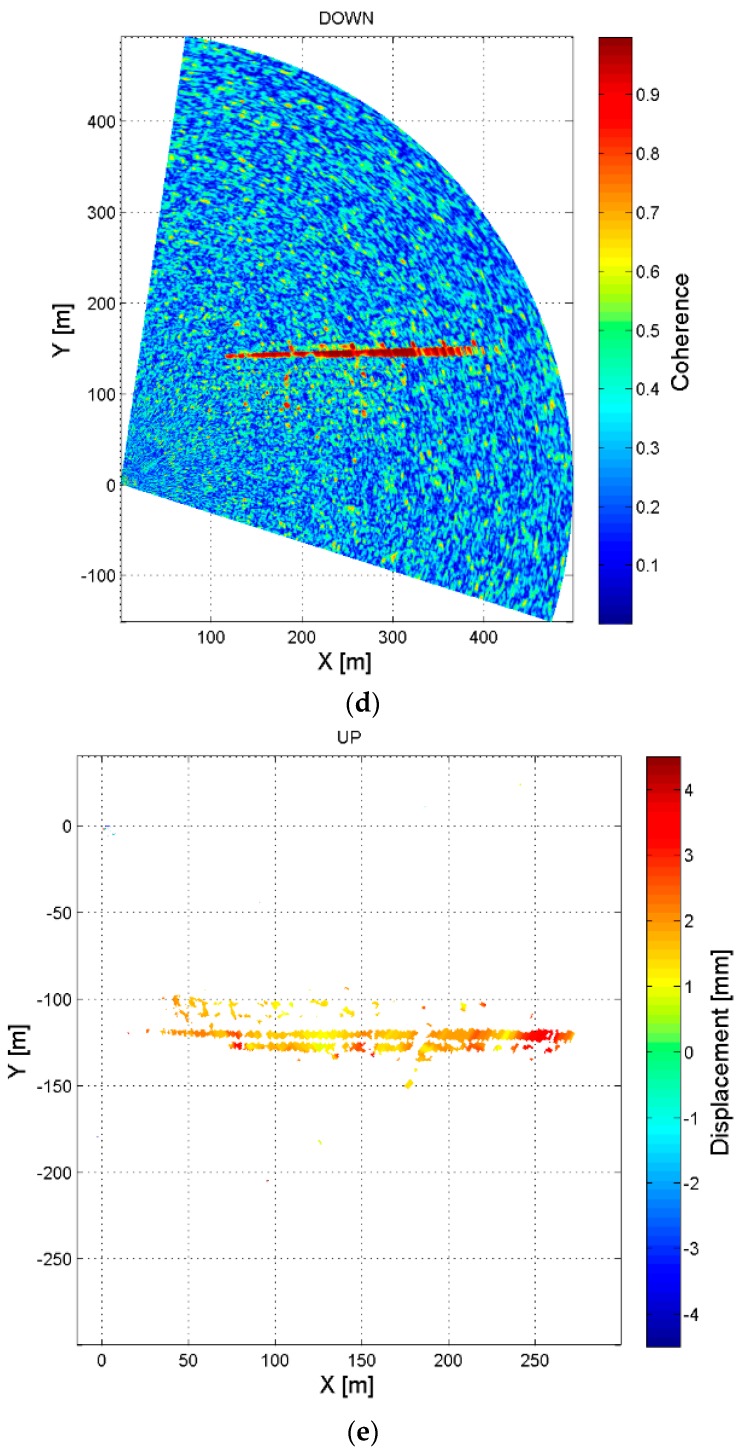

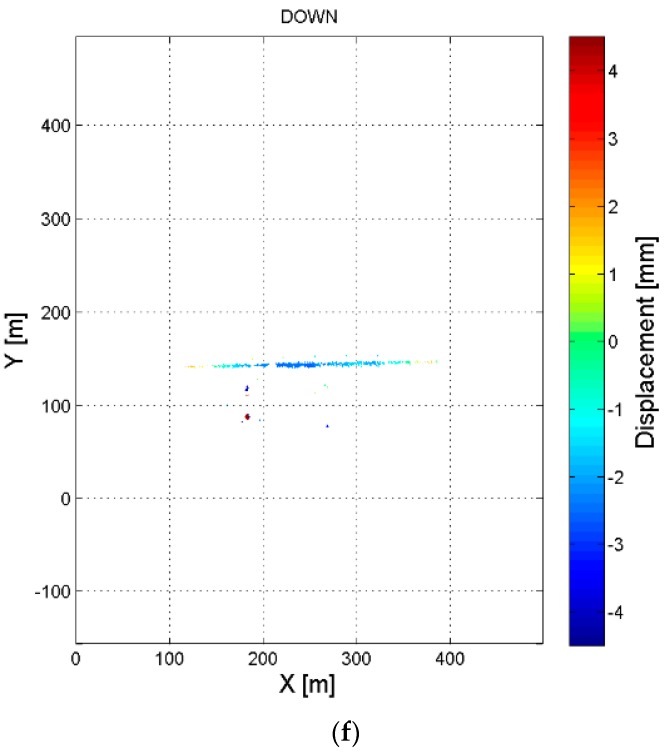
San Pietro dam, Aquilonia. (**a**,**b**) photo of the dam as observed from the GBSAR positions; (**c**,**d**) interferometric coherence; (**e**,**f**) displacement maps in Cartesian coordinates. Data have been collected by two GBSAR systems installed on upper (UP) and downstream (DOWN) sides of the dam, respectively.

Figure 12 summarizes the results that have been obtained at the San Pietro dam. Two sites have been used for the installation of the GBSAR, on the downstream and upstream sides of the dam (see Figure 12a,b). The coherence and displacement maps refer to data acquired on 11 September and 15 November 2014 (see Table 1). The temporal baseline of about two months partially decorrelates the radar signal scattered from the upstream surface, due to the rise of water level. The measured displacements vary between 1 and 2 mm on the upstream surface and between 1 and 2 mm on the downstream surface. A positive displacement means a movement away from the radar. In this case, the opposite signs of displacement measured can be easily interpreted in terms of the increased water level registered between the first and the second measurement campaigns. This particular acquisition geometry can be useful to detect different displacement patterns on the downstream and upstream surfaces and verify that the earth dam behaves as a rigid body.

### 5.2. Visualization of Displacements and Vibration Frequencies of Concrete Dam Structures

**Figure 13:**
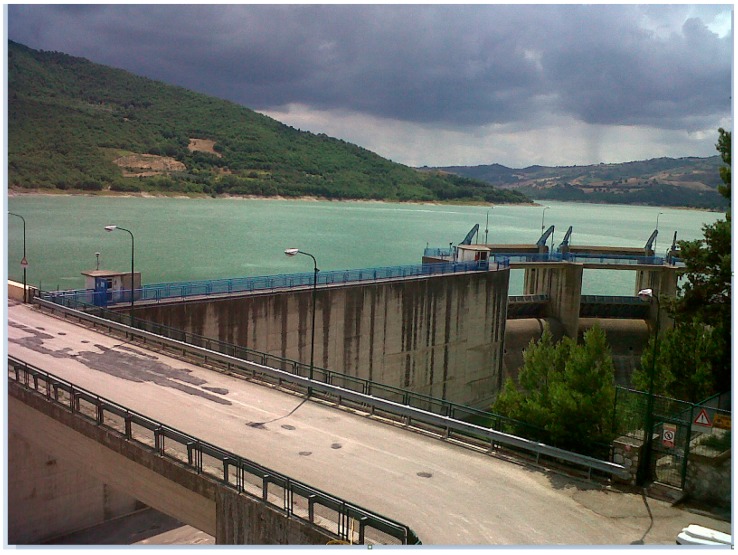
Occhito dam, Carlantino. Monitoring of the footbridge.

**Figure 14:**
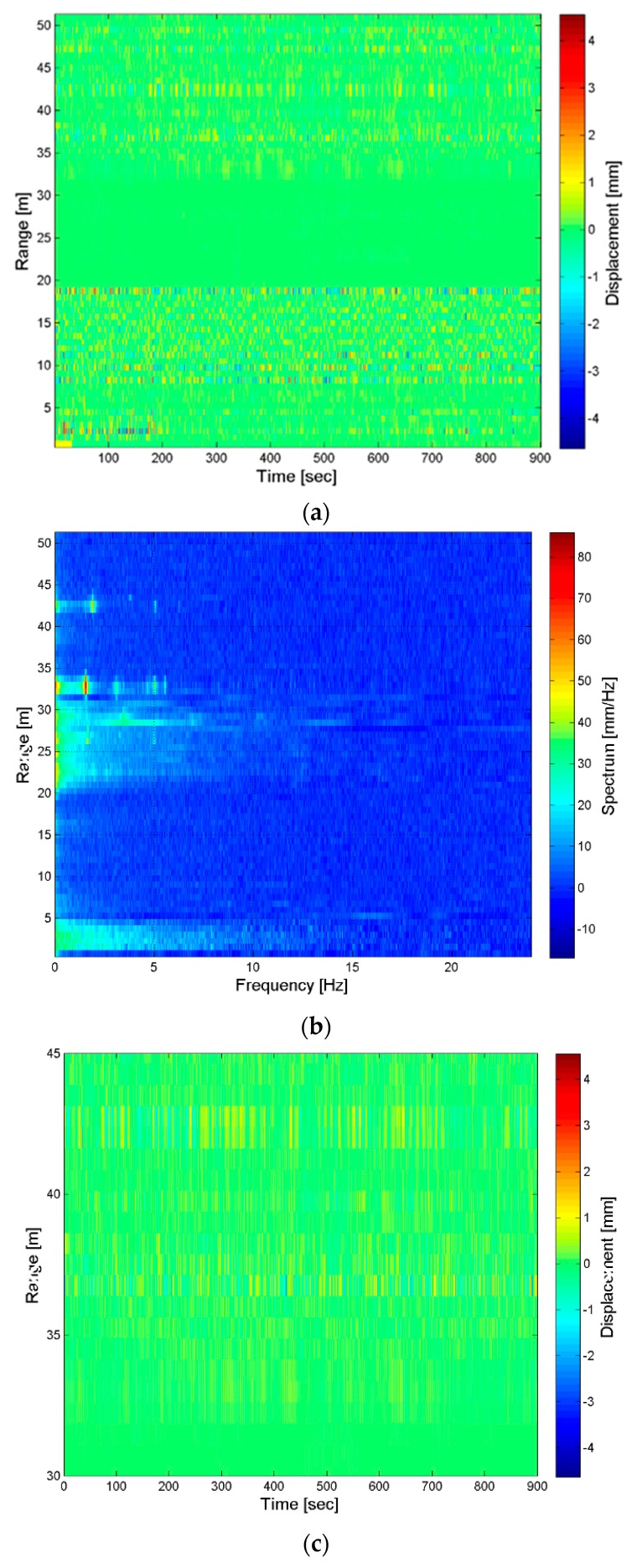

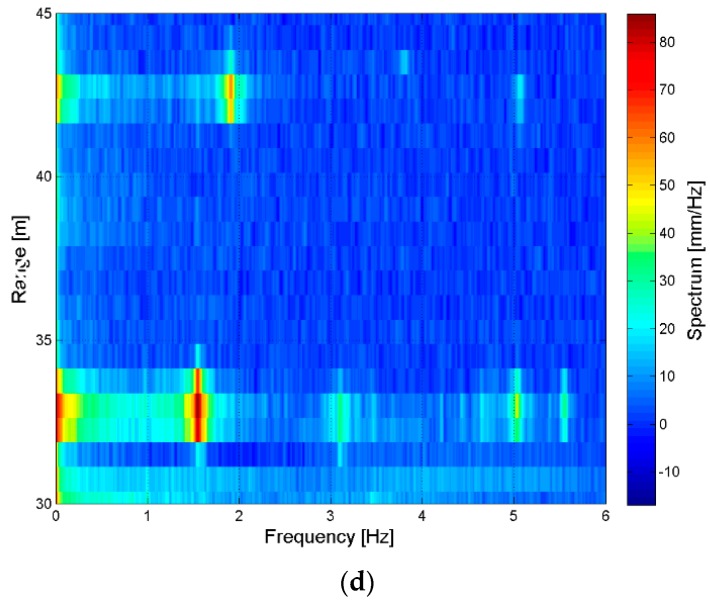
Occhito dam, Carlantino. Monitoring of the pedestrian bridge. Holistic view of (**a**) displacements and (**b**) frequency spectrum of all targets observed by the GBSAR. Frequency peaks can be observed in the range distance intervals [30 m, 35 m] and [40 m, 45 m]. Details of the (**c**) displacement and (**d**) frequency spectrum with the peaks at 1.6 Hz and 2 Hz.

This section shows the measurements of displacements and vibration frequencies of concrete structures being parts of the earth dam infrastructures are shown. The measurement of these physical quantities has been carried out by means of a RAR device. Time series of displacements of targets located at different range distances are represented as a map. The main advantage of this representation is to facilitate the identification of common displacement features and to discriminate spurious signals, due to atmosphere, or to identify displacement features of other targets in the scene, not belonging to the dam structure. A similar representation is used also for the vibration frequencies, with a map generated by stacking all profiles of amplitude spectrum vs. the vibration frequency for targets located at increasing range distances. Figure 13 and Figure 14 refer to measuring the monitoring of the pedestrian bridge, part of the Occhito dam. The RAR has been installed at the foot of the bridge, illuminating the bridge deck. The maps with the stacked frequency spectra shown in Figure 14 help to identify two frequency peaks, one of 1.6 Hz observed in correspondence of targets located in the range interval [30 m, 35 m], the other of 2 Hz for target in the range interval [40 m, 45 m]. 

**Figure 15:**
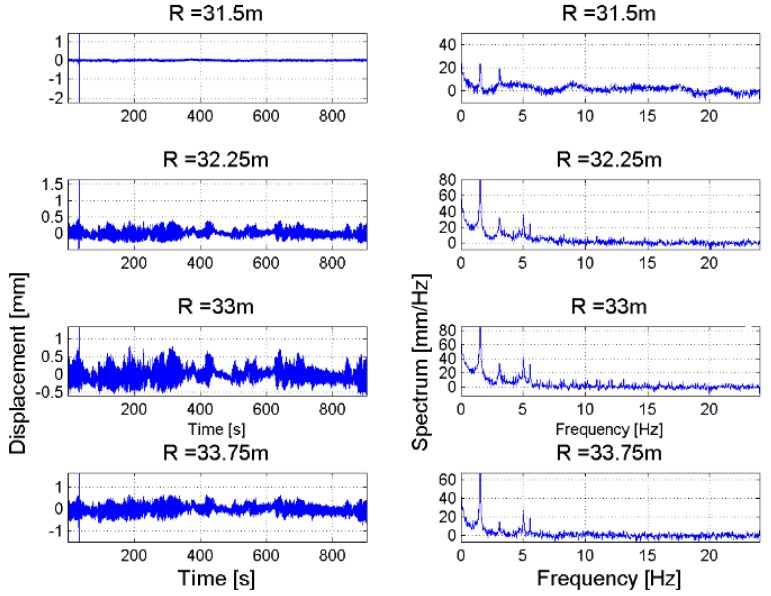
Occhito dam, Carlantino. Time series of displacements (**left**) and corresponding frequency spectrum (**right**) of targets belonging to the pedestrian bridge located at range distance in the interval [30 m, 35 m].

**Figure 16:**
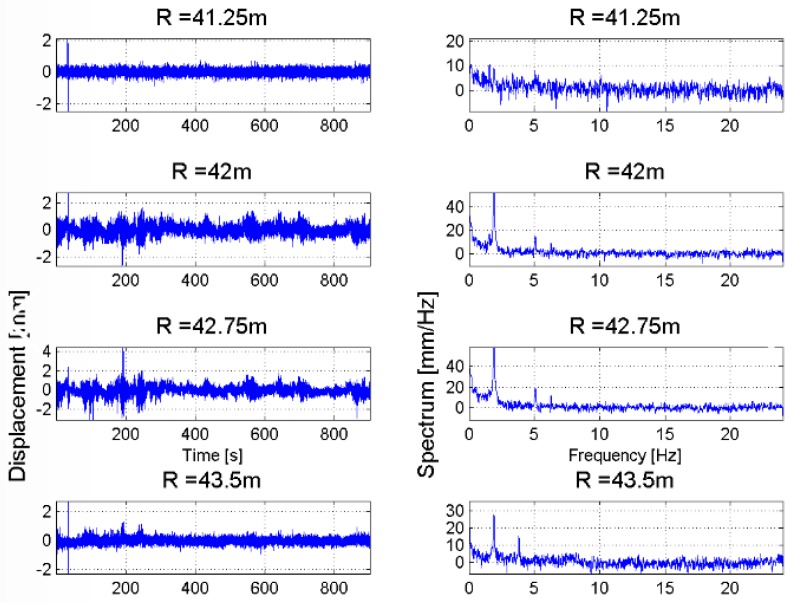
Occhito dam, Carlantino. Time series of displacements (**left**) and corresponding frequency spectrum (**right**) of targets belonging to the pedestrian bridge located at range distance in the interval [40 m, 45 m].

Details about the displacement profiles and the corresponding spectra for targets located in the two above range interval are reported in Figure 15 and Figure 16. The maximum measured displacement was less than 1 mm. It is worth noting that, while the measured LoS displacement depends on the observation geometry, i.e., the relative orientation between the main axis of the structure and the antenna main lobe, the frequency spectrum does not change when modifying the observation geometry, except when the radar LoS direction is perpendicular to the structure. In fact, in this case the measurement becomes extremely noisy.

**Figure 17:**
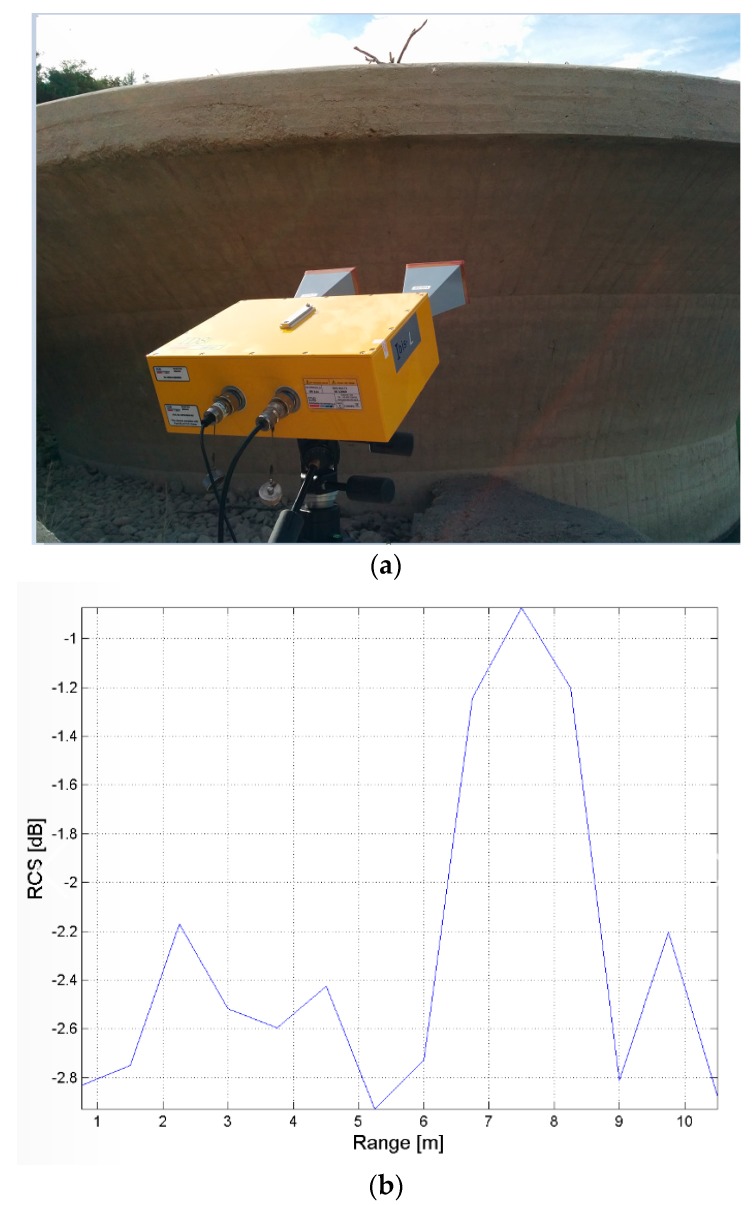
San Pietro dam, Aquilonia. Monitoring of the chalice-shaped spillway. (**a**) observation geometry with detail of the antennas used for the data acquisition; (**b**) Radar cross-section of the radar signal scattered by the structure vs. the range distance from the radar.

**Figure 18:**
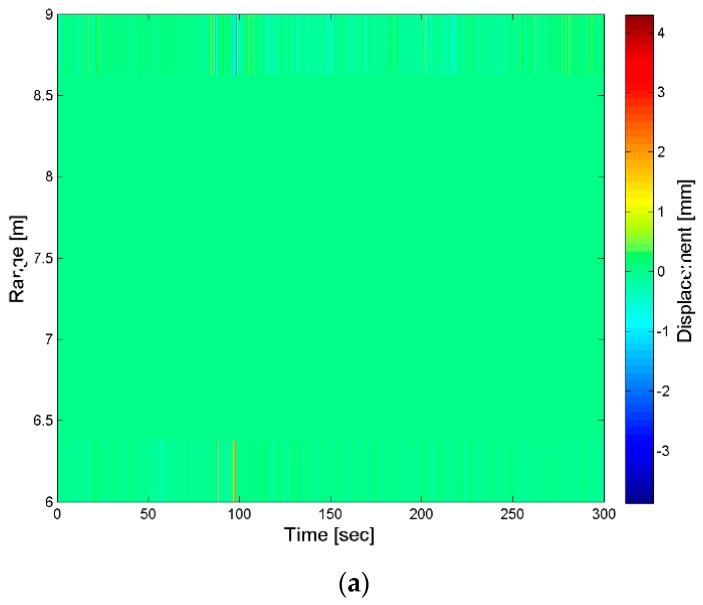

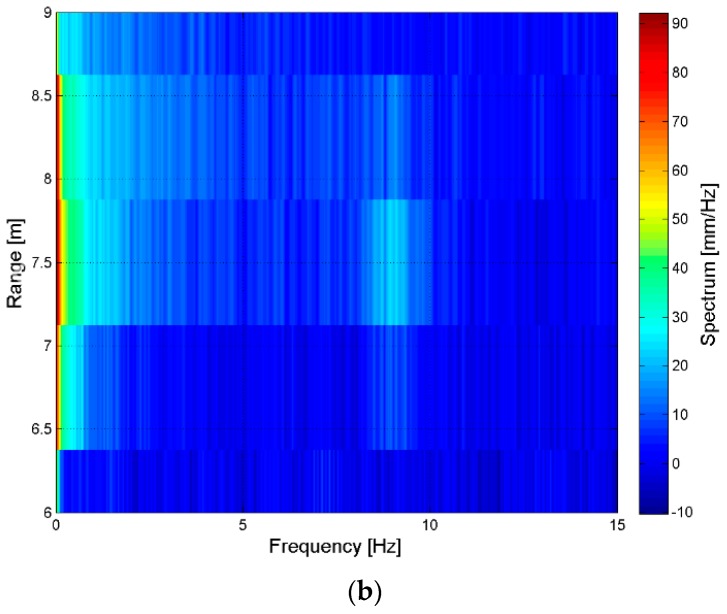
San Pietro dam, Aquilonia. Monitoring of the chalice-shaped spillway. Maps with (**a**) displacement time-series and (**b**) vibration spectrum of targets located at range distances between 6 and 9 m.

**Figure 19:**
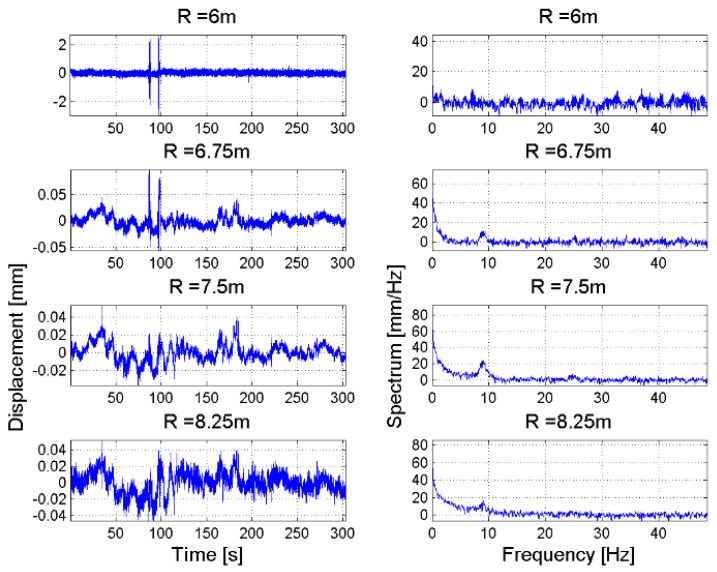
San Pietro dam, Aquilonia. (**left**) time series of displacements; (**right**) corresponding frequency spectrum of targets belonging to the chalice-shaped spillway located at range distance in the interval [5 m, 10 m].

The visualization tool is applied to the data acquired for the monitoring of the chalice-shaped spillway of the San Pietro dam. In this case, the GBSAR has been installed at a distance of a few meters from the concrete structure (see Figure 17a). The RCS profile reported, in Figure 17b, shows that the chalice-shaped spillway is located a distance between *R* = 5 m and *R* = 8 m from the radar as in this region the highest response from the scene is observed, due to the backscattering of the concrete structure. Outside this range interval, the radar response is basically due to the atmosphere and it is characterized by a RCS lower than at least 25 dB. Figure 18 shows the map with the stacked displacement profiles vs. time and amplitude spectra vs. vibration frequencies maps. A small peak in correspondence of the vibration frequency of *f* = 9 Hz was observed for targets located between *R* = 7 m and *R* = 8 m from the RAR. For these targets a displacement smaller than 0.5 mm has been measured as shown in Figure 19, which reports also the corresponding spectra.

The two examples of the pedestrian bridge and chalice-shaped spillway describe the way to use the visualization tool of RAR data. As a first step, the maps of stacked displacements and vibration frequencies should be used to identify regular patterns in the displacements of targets located in the scene or specific frequency peaks. As a second step, the displacement profiles and vibration spectra of the targets of interest, identified at first step, are analyzed in detail.

## 6. Conclusions

In this paper, we have presented a protocol to acquire and process GBSAR and RAR measurements for the monitoring of earth dams. The advantages of using two GBSAR systems, properly located with respect to the dam, have been shown both in terms of more effective visualization and quantitative analysis of dam displacements. Two methodologies have been described. The former corrects LoS displacements providing the real dam displacement vectors, geolocating the maps and rendering them on the dam digital surface representation. The latter analyzes RAR measurements of concrete structures to measure their displacements and visualize the corresponding frequency spectrum. Both the protocol and the visualization tools can help structural engineers to routinely use a ku-band ground-based radar for a non-destructive seismic assessment of earth dams in any weather condition and also in absence of sun illumination. In particular, the two methodologies presented in this paper can be used by structural engineers to have geolocated displacement vectors over both downstream and upstream dam surfaces and displacement time series, with the corresponding vibration spectra, of all the elements of the concrete structure. As a consequence, spatially dense displacement information will be available for the numerical modeling of the whole dam infrastructure. This could open new perspectives for the dam modeling during seismic events if continuous GBSAR measurements of the dam surface and its concrete structures are acquired, at least for the larger and most important dams located in seismic prone areas.

## Figures and Tables

**Table 1:** Earth dam characteristics and acquisition dates (L = SAR modality, S = RAR modality).

Earth Dam	Height (m)	Crown Length (m)	Capacity (mcm)	Acquisition Dates
Occhito	60.40	432	247.50	27/05/2013 (L)
				27/06/2013 (L, S)
				02–03/03/2014 (L)
				12/09/2014 (L)
Capaccio	24.30	3290	16.8	06/11/2013 (L)
				21/01/2014 (L)
Marana-Capaciotti	50	825	48.20	07–11/11/2013 (L)
				25/01/2014 (L)
San Pietro	49	450	14.5	30/10/2013 (L)
				15/01/2014 (L)
				11/09/2014 (L, S)
